# Supplementary material for: Heat conduction measurements in ballistic 1D phonon waveguides indicate breakdown of the thermal conductance quantization
Source: Nat Commun. 2018 Oct 16;9:4287. doi: 10.1038/s41467-018-06791-0 (PMC6191430; doi:10.1038/s41467-018-06791-0)
Supplement: Supplementary file 1 — Supplementary Information [file 41467_2018_6791_MOESM1_ESM.pdf]

Heat conduction measurements in ballistic 1D phonon waveguides indicate breakdown of the thermal conductance quantization

Tavakoli et al.

Supplementary Information

Supplementary Note

Numerical simulations of transmission coefficients of contacts having different sizes

In previous modelling [1,2] the contacts have always been considered as semi-infinite constant-thickness wires. By doing this, it was found that a catenoidal-shaped constriction yields a good thermal contact with little backscattering. However, there is no clear reason to assume that the infinitely extended wire model is a fair representation of the actual experimental contact. The very large (macroscopic) mean free paths and coherence lengths of phonons at 1K temperatures mean that other features away from the catenoidal constriction might affect and partly block the single phonon modes propagating through this constriction.

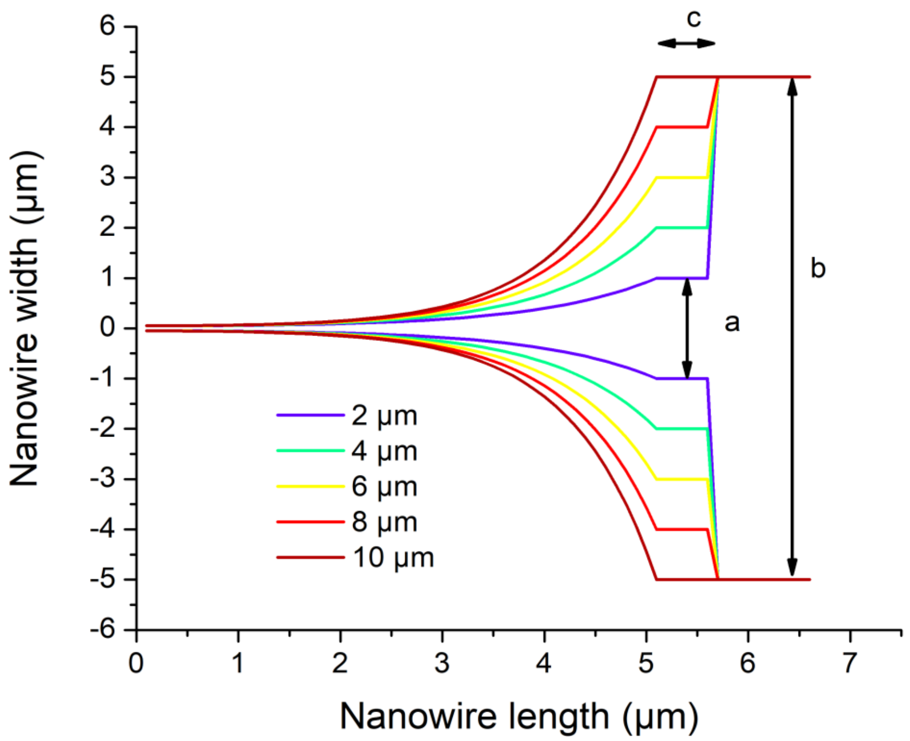

Supplementary Figure 1. ***Geometry of the constriction between nanowire and reservoir.*** *The geometry of the nanowire and the connection to the contact for various sizes of constriction* **a** *(see colour scheme) between the nanowire and the contact of width* **b***=10 µm;* **c** *being the size of the straight segment. In the present experiment* **a** *is equal to 2.7 µm for the results presented in Fig. 3 in the main text and equal to 2 µm for the Fig. 4.*

Using a scalar model with only dilatational displacements, similar to Rego and Kirczenow's [1], it is easy to see a strong reduction in transmission if the

catenoidal constriction is abruptly plugged into a much thicker contact. Supplementary Figure 1 sketches five profiles for a single contact, with dimensions similar to those in the present experiment (lengths are given in micrometers). The transmission probability has been calculated for a single contact using Green's functions, assuming a sound velocity of 10 km/s for the dilatational mode. This transmission for each of the profiles is plotted as a function of phonon frequency. We have considered 100 nm for the thickness left-hand-side contact, a length of 5 µm for the catenoidal junction, and **c** = 500 nm for the length of the straight segment joining the end of the catenoidal part (with thickness **a**) to the beginning of the thick contact (with thickness **b** = 10 µm). The calculated transmission (Supplementary Figure 2) shows that if **a** decreases with respect to **b**, the transmission also decreases leading to a reduction of thermal conductance as it is seen in the present experiment. **a** is equal to 2.7 µm for the results presented in Fig. 3 in the main text and equal to 2 µm for the Fig. 4.

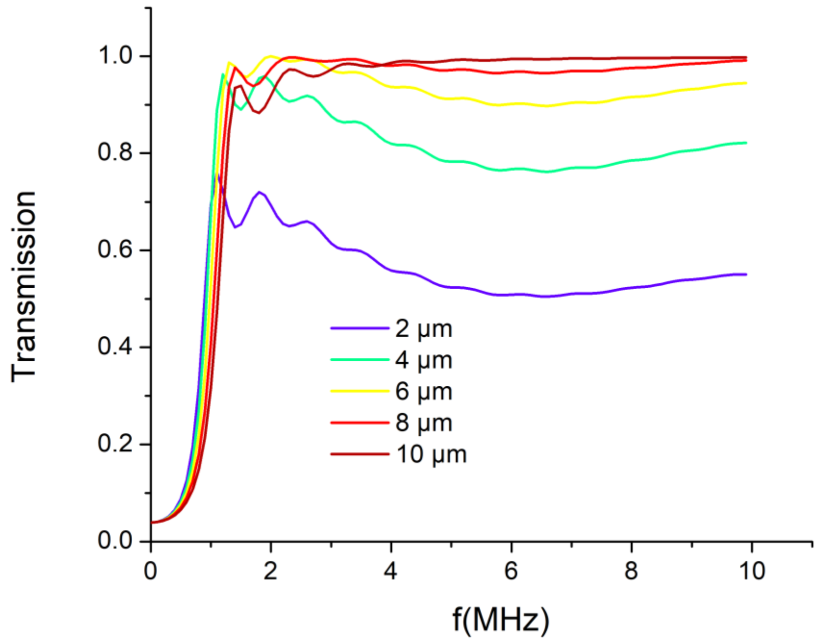

Supplementary Figure 2. ***Transmission coefficients for various constriction sizes.*** *The transmission probability as a function of phonon frequency is calculated using Green's function technique for each size of constriction* **a** *(from 2 µm to 10 µm).*

Such a one dimensional scalar model is however not a fair representation of the true transmission, because it does not allow for vibrations to scatter into modes other than the *x*-dependent dilatational one. This limitation is inherent to Rego and Kirczenow's model too [1]. Tanaka *et al.* considered polarizations other than

the dilatational one, however the results presented in that paper are also restricted to infinitely extended wire-type contacts [2]. In order to properly take into account the true structure, a fully three dimensional model, considering displacements in all three spatial dimensions, and allowing for waves to depend also on three dimensions, would need to be implemented and applied to the real structure. Such is a complex undertaking and we are unaware of any published results on systems similar to the one measured here.

This rough model is therefore just qualitative, and it is only provided to illustrate the fact that large structural features away from the constriction can lead to a reduced transmission. Only a fully 3D large-scale calculation can provide a quantitative picture.

Supplementary References

[1] Rego, L.G.C. and Kirczenow, G. Quantized thermal conductance of dielectric quantum wires. *Phys. Rev. Lett.* **81**, 232-235 (1998).

[2] Tanaka, Y., Yoshida, F., and Tamura, S. Lattice thermal conductance in nanowires at low temperatures: Breakdown and recovery of quantization. *Phys. Rev. B* **71**, 205308 (2005).
